# Interplays of glucose metabolism and KRAS mutation in pancreatic ductal adenocarcinoma

**DOI:** 10.1038/s41419-022-05259-w

**Published:** 2022-09-24

**Authors:** Yu-Huei Liu, Chun-Mei Hu, Yuan-Sheng Hsu, Wen-Hwa Lee

**Affiliations:** 1 Drug Development Center, China Medical University, Taichung, Taiwan; 2 Graduate Institute of Integrated Medicine, China Medical University, Taichung, Taiwan; 3 Department of Medical Genetics and Medical Research, China Medical University Hospital, Taichung, Taiwan; 4 Graduate Institute of Biomedical Sciences, China Medical University, Taichung, Taiwan; 5 Genomics Research Center, Academia Sinica, Taipei, Taiwan; 6 Department of Biological Chemistry, University of California, Irvine, CA USA

**Keywords:** Cancer metabolism, Biochemistry

## Abstract

Pancreatic ductal adenocarcinoma (PDAC) is one of the most aggressive and deadliest cancer worldwide. The primary reasons for this are the lack of early detection methods and targeted therapy. Emerging evidence highlights the metabolic addiction of cancer cells as a potential target to combat PDAC. Oncogenic mutations of *KRAS* are the most common triggers that drive glucose uptake and utilization via metabolic reprogramming to support PDAC growth. Conversely, high glucose levels in the pancreatic microenvironment trigger genome instability and *de novo* mutations, including *KRAS*^*G12D*^, in pancreatic cells through metabolic reprogramming. Here, we review convergent and diverse metabolic networks related to oncogenic *KRAS* mutations between PDAC initiation and progression, emphasizing the interplay among oncogenic mutations, glucose metabolic reprogramming, and the tumor microenvironment. Recognizing cancer-related glucose metabolism will provide a better strategy to prevent and treat the high risk PDAC population.

## Facts

- Although the association between diabetes and PDAC has been revealed, whether diabetes is a predisposing factor or an early manifestation of malignancy remains unsettled.
- One of the potential therapeutic strategies for PDAC is to target the metabolic addiction of cancer cells.
- *KRAS* proto-oncogene mutations shut glycolysis hexosamine biosynthesis and pentose phosphate pathways.
- High glucose initiates genome instability and *de novo* mutations, including *KRAS*^*G12D*^, in nontumorigenic pancreatic cells.
- Alternation of O-linked-N-acetylglucosaminylation changes cellular and physiological homeostasis fueling PDAC initiation and progression.

## PDAC: a growing silent killer

Pancreatic ductal adenocarcinoma (PDAC), the seventh leading cause of cancer-related death worldwide in 2020, accounts for ~95% of all pancreatic cancers as well as 4.9% and 4.5% of estimated age-standardized incidence and mortality rates, respectively, with almost as many deaths as the number of cases [1]. PDAC will foreseeably become the second leading cause of cancer-related deaths by 2026 [2], with an ~11.5% 5-year relative survival rate in the United States [3]. Due to the lack of an early detection method and effective therapeutics, diagnosis generally occurs at an advanced stage, when patients already have locoregional extensions or metastases that render surgical resection ineffective [4]. PDAC stems from abnormal acinar-to-ductal metaplasia (ADM) and pancreatic intraepithelial neoplasia (PanIN) of grades I–III [5]. One of the potential therapeutic strategies for PDAC is to target the unique nutrient availability and utilization in cancer cells [6, 7]. Significant advances have been made in understanding metabolic adaptations to *KRAS* hyperactivation. Efforts are ongoing to design metabolism-targeted diagnostic and therapeutic strategies. Nevertheless, the most potent strategy should aim to prevent PDAC initiation and enhance early detection.

Considering genetic and personal risk factors [8], novel findings have suggested aberrant metabolites not only as promoters [9] but also as initiators [10] for PDAC, and common metabolic reprogramming patterns have gained a hotspot for prevention or early detection [11]. This review provides a synopsis of metabolic dependence supporting oncogenic mutation-driven PDAC progression, emphasizing that aberrant nutrient availability and utilization may also cause oncogenic mutations. This review also offers an outlook of potential targets for PDAC therapeutics, prevention, or early detection.

## *KRAS* mutation and metabolic alterations

Cancer cells become dependent on activated oncogenes or their downstream metabolic processes for survival and proliferation [12]. Inhibiting oncogenes or their downstream mediators is expected to be lethal to metabolically addicted cells without harming normal cells. Advances include dependence on poly(ADP-ribose) polymerase activity in the context of *BRCA* deficiency [13, 14]. The somatic mutation of oncogenic *KRAS* is the most recognized genetic alteration and is thus the most attractive drug target in PDAC [15, 16]. Mutant KRAS increases the expression of glucose transporter 1 (GLUT1) and rate-limiting glycolytic enzymes, including hexokinases, phosphofructokinase 1 (PFK1), and lactate dehydrogenase A (LDHA), promoting glycolytic activity and increasing lactate production [17, 18]. By upregulating these enzymes, mutant KRAS triggers the shunting of glycolytic intermediates into the hexosamine biosynthesis pathway (HBP) to generate UDP-N-acetylglucosamine (UDP-GlcNAc) for glycoprotein, glycolipid, proteoglycan, and glycosylphosphatidylinositol anchor biosynthesis in cancer cells [5, 19]. The shunting of glycolytic intermediates into the nonoxidative arm of the pentose phosphate pathway (PPP) generates the ribose 5-phosphate necessary for nucleic acid biosynthesis and nicotinamide adenine nucleotide phosphate (NADPH) for regenerating glutathione (GSH) from oxidized GSH (GSSG) to support ROS scavenging for redox balance. GSH biosynthesis depends on glutamine. While most cells convert glutamine-derived glutamate to α-ketoglutarate via glutamate dehydrogenase (GLUD1) to fuel the tricarboxylic acid (TCA) cycle, PDAC relies on glutamine-derived aspartate via glutamic oxaloacetate transaminase 1 (GOT1), which is catalyzed by the mitochondrial uncoupling protein 2 (UCP2) [20]. Glutamine-derived aspartate can be converted into oxaloacetate, which is subsequently converted into malate and then pyruvate, increasing the NADPH/NADP^+^ ratio, which could additionally maintain the cellular redox balance for PDAC progression [21]. Other metabolic adaptations to *KRAS* hyperactivation include the dependence of amino acids such as serine [21, 22], glycine [23], branched-chain keto acid [24, 25], and lipid metabolism [26] in PDAC to facilitate survival [16, 27], which mirrors genetic heterogeneity in PDAC [28–30], are summarized in BOX1. Efforts on designing suitable metabolism-targeted diagnostic and therapeutic strategies are ongoing.

**Fig. 1:**
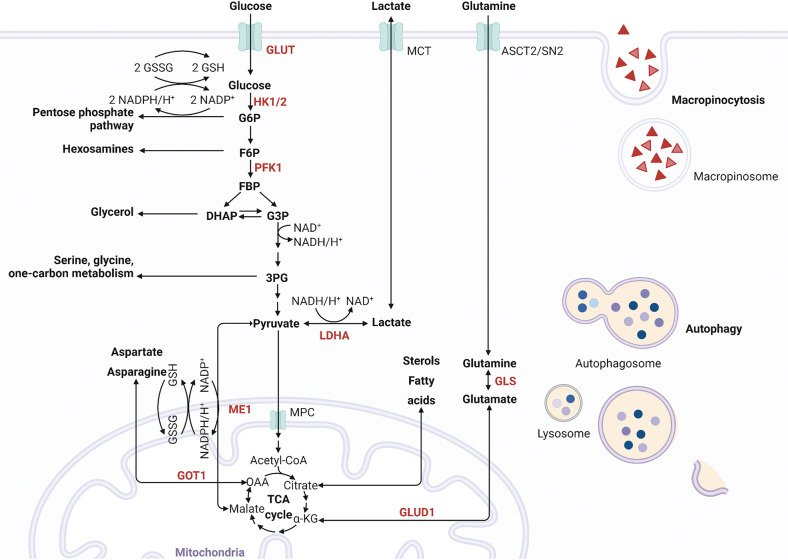
Mutant KRAS mediated metabolic alterations. The active oncogenic KRAS mutant triggers glycolysis for energy generation and building block biosynthesis and shunting into the nonoxidative arm of the pentose phosphate pathway for nucleic acid biosynthesis and the hexosamine biosynthesis pathway for UDP-N-acetylglucosamine biosynthesis through upregulation of key enzymes in glycolysis. In addition, micropinocytosis and autophagy ensure that mutant KRAS cancer cells obtain energy and biosynthetic building blocks from the microenvironment. GLUT1 glucose transporter 1, HK1/2 hexokinases 1 and 2, PFK1 phosphofructokinase 1, LDHA lactate dehydrogenase A, ASCT2, also known as solute carrier family 1, member 5 (SLC1A5); SN2, also known as solute carrier family 38, member 5 (SLC38A5); MCT monocarboxylic transporters, GOT aspartate transaminase, GLUD1 glutamate dehydrogenase 1, GLS1 glutaminase 1, G6P glucose 6-phosphate, F6P fructose 6-phosphate, FBP fructose 1,6-phosphate, DHAP dihydroxyacetone phosphate, G3P glyceraldehyde 3-phosphate, 3PG 3-phosphoglycerate, αKG α-ketoglutarate, Asp aspartate, OAA oxaloacetate, TCA cycle tricarboxylic acid cycle. The graph was created with BioRender.com.

In addition, nutrient deprivation in the PDAC tumor microenvironment (TME) [31] may drive *KRAS*-mutated cancer cells to be more dependent on lysosomal nutrient scavenging pathways, such as macropinocytosis [27] and autophagy [32], to recycle energy and biosynthetic building blocks from the microenvironment [16]. Inactivating oncogenic *Kras*^*G12D*^ in an inducible *Kras*^*G12D*^ murine model of PDAC reversed metabolic reprogramming and rapid proliferation, supporting that *Kras* mutation is essential for PDAC initiation and progression [18, 33–35]. Figure 1 illustrates the current understanding of metabolic addictions driven by *KRAS* mutations. However, withdrawal of transgene expression may have differential effects from the loss of endogenous oncogenic *KRAS* expression caused by inhibitors. PDAC cells survive even genetic ablation of *Kras* both in vitro and in vivo [36, 37], indicating that reversing the oncogenic behaviors driven by mutant *KRAS* might not completely halt all PDAC development. Additional *non-KRAS* genetic heterogeneity and other genetic alterations may dysregulate metabolism in PDAC, which are summarized in BOX 1. However, how the polygenic risk of the mutations that shape the metabolism and phenotype of cancer cells remains to be elucidated [38].

### Association between diabetes and PDAC

The association between diabetes and PDAC has been recognized. The prevalence of diabetes is higher in PDAC than in other cancers [11]. Diabetic patients with PDAC frequently have larger tumors, reduced median survival [39], and higher mortality [40, 41]. Unlike insulin and proinsulin levels, which are independently associated with an increased risk of PDAC among nondiabetic patients, circulating markers, including baseline fasting blood glucose and glycated hemoglobin, are associated with the increased risk of PDAC a dose-dependent manner among patients with diabetes [42, 43]. However, ~45–65% of patients with PDAC have diabetes at diagnosis, with new-onset diabetes (within <3 years) accounting for ~25% [44], implying that hyperglycemia is not essential for PDAC development. Although whether diabetes is an early manifestation of malignancy [11, 45] or a predisposing factor for the development of PDAC [46–48] is unsettled, the association of diabetes with PDAC has gained sufficient support from different studies.

### High glucose-triggered DNA damage and de novo mutations, including *KRAS*^*G12D*^_,_ drive proliferating acinar cell transformation

Oncogenic *KRAS* mutations are thought to be an early genetic mutation that exerts dramatic metabolic reprogramming in PDAC. However, oncogenic *KRAS* mutations are not sufficient to initiate PDAC [49, 50]. Other factors are required to orchestrate tumor formation. The physiological roles of the pancreas are in modulating carbohydrate, protein, and lipid metabolism, as well as the availability and utilization of metabolites such as lactate and glutamine [51, 52]. Interestingly, glucose is not used as the primary energy source for the pancreas, unlike other organs [51, 52], implying that excess metabolites such as glucose may be a risk factor for PDAC initiation [53]. Oncogenic *Kras* and a chronic high-fat diet synergistically promote PDAC development [9]. Although with a lower tumorigenic capacity than a chronic high-fat diet, a chronic high-carbohydrate diet promoted ADM and PanIN lesions with increased inflammation and fibrosis compared to a normal diet in *Kras*^*G12D/+*^ mice [54]. In contrast, a low glycemic diet impairs tumor growth by altering cellular lipid composition [55]. Sugar consumption contributes to the development of metabolic diseases, including type 2 diabetes [56, 57], and some cancers, partly through weight gain but also through independent metabolic effects of glucose and fructose [58]. Epidemiological studies have highlighted a potential association between sugar consumption and PDAC incidence and mortality [59–61]. However, it remains inconclusive in part because of the limitations of study design and evaluation tools to appropriately weigh the strength of genetic predisposition- or sugar consumption-induced obesity and metabolic outcomes [58, 60, 61].

Comparing nonmalignant pancreatic samples from PDAC individuals with and without a history of diabetes revealed that pancreases with diabetes sustained significantly more DNA damage and DNA mutation than other organs, suggesting a pancreas-specific effect [10]. Acinar cells or nontumorigenic pancreatic cells in long-term high glucose culture increased the average *KRAS* mutation rate, although to a small extent, supporting the selective advantage of glucose for *KRAS* hotspot mutations [10]. The increases in the *de novo KRAS*^*G12D*^ mutation rate were consistent regardless of the different measurement tools, including immunofluorescence staining and flow cytometry followed by sequencing. The lack of a significant increase in pyruvate/lactate production under high glucose in nontumorigenic pancreatic cells under aerobic conditions supported that metabolic reprogramming is not energy-consuming [62]. The increase in unscheduled glycolysis may lead to DNA damage at least partially by reducing the dNTP pool by O-GlcNAcylation of ribonucleotide reductase catalytic subunit M1(RRM1), which may subsequently cause adaptive DNA mutations to maintain cell survival [10].

In addition, since *KRAS* mutation alone is insufficient to generate PDAC [49, 50], specific pancreatic cells containing *KRAS* mutations may require additional alterations to exhibit subtle phenotypic changes at the beginning. It remains to be explored whether metabolic factors in the glucose pathway and microenvironmental nutrients may play key roles in this regard.

## Glycosylation and KRAS mutation

O-linked-N-acetylglucosaminylation (O-GlcNAcylation) is a unique nutrient- and stress-responsive glycosylation that involves the addition of N-acetylglucosamine (GlcNAc) moieties from UDP-GlcNAc to serine and threonine residues of proteins. The addition and removal of *O*-GlcNAc rely on *O*-GlcNAc transferase (OGT), which adds UDP-GlcNAc to target proteins, and *O*-GlcNAcase (OGA), which removes *O*-GlcNAc from O-GlcNAcylated proteins [63]. The generation of UDP-GlcNAc is not only for O-GlcNAcylation but also for O-glycosylation and N-glycosylation of proteins to maintain cellular survival under stress [63]. As a nutrient sensor, HBP is essential for amino sugar biosynthesis by integrating core metabolic intermediates from glycolysis, fatty acids, amino acids, and nucleotide metabolism [64, 65]. However, either cancer cells under glucose deprivation, hypoxia, or oncogenic KRAS mutation [8] or nontumorigenic pancreatic cells under high glucose levels [10] cause hyper-O-GlcNAcylation of cellular proteins, challenging the regulation of O-GlcNAcylation via the versatile UDP-GlcNAc.

Metabolic reprogramming may be dynamic, unsynchronized, and coordinated with neighboring cells in an organ in response to extracellular and intracellular stresses. Several O-GlcNAcylation targets related to PDAC metabolism and progression, such as notch receptor 1 promoting cancer development [66], SRY-box transcription factor 2 regulating self-renewal [67], sirtuin 7 (SIRT7) triggering cancer progression by blocking the SIRT7-proteasome activator subunit 3 (PAME3) interaction [68], and nuclear factor kappa B modulating cancer-associated inflammation [69], have been reported. Although hyper-O-GlcNAcylation of insulin receptor substrate 1 and AKT serine/threonine kinase 2 inhibits their phosphorylation and induces insulin resistance in peripheral cells [70], the corresponding regulation of endocrine and exocrine secretion from the pancreas remains unclear. Protein O-GlcNAcylation in response to high glucose-driven metabolic reprogramming may potentially have multiple targets beyond PFK1 and RRM1 in nontumorigenic pancreatic cells [10]. The precise control and crucial targets of O-GlcNAcylation and how O-GlcNAcylation helps adapt and maintain homeostasis in a specific organ under stress remain to be explored. The safe use of glucosamines, the UDP-GlcNAc precursor, as a dietary supplement for osteoarthritis under variable O-GlcNAcylation due to UDP-GlcNAc imbalance or OGT/OGA dysregulation has to be reconsidered [62, 71].

Thus, unlike glycated proteins such as carbohydrate antigen 19-9 (CA19-9) and hemoglobin A1c (Hb A1c) with mutually correlated levels [72], glycosylated proteins that help cancer cells adapt to stress and malignant phenotypes could serve as potential diagnostic and therapeutic targets [63, 71].

### Potential factors regulating HBP in a high glucose status

HBP is essential for versatile UDP-GlcNAc synthesis. Regulations of enzymes critical for HBP and controlling high glucose-induced protein O-GlcNAcylation should be discussed. PFK1, a 340 kDa heterotetrameric allosteric enzyme composed of PFKL, PFKM, and PFKP, works with a concerted symmetric transition from an enzymatically inactive T-state to the active R-state and can be dissociated into inactive dimers and monomers [73]. Increased PFK activity may help pancreatic cells maintain their survival advantage during adaptation to the poor oxygen and nutrient supply microenvironment [18]. In contrast, high glucose-induced O-GlcNAcylation inactivates PFK1 and RRM1 and subsequently inhibits glycolysis and dNTP generation, respectively, resulting in high-frequency DNA damage, specifically in pancreatic cells [10]. Modulation of PFK activity during adaptation or hyperproliferation may result from different posttranslational modifications and/or subunit assembly, which warrants further investigation.

**Fig. 2:**
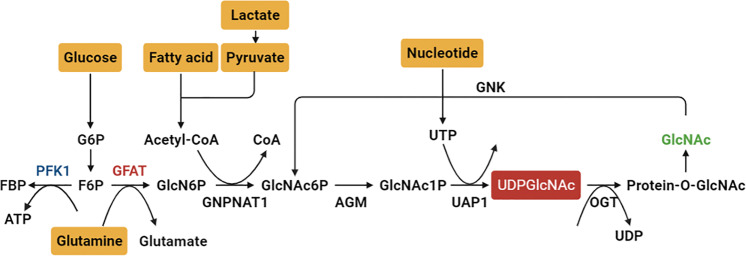
The *de novo* hexosamine biosynthetic pathway (HBP) and GlcNAc salvage pathway integrate metabolic status from core metabolism intermediates, including glycolysis, fatty acid, amino acid and nucleotide metabolism, to generate uridine diphosphate-N-acetylglucosamine (UDP-GlcNAc). Approximately 2–5% of cellular glucose enters the HBP to generate the end product UDP-GlcNAc. Glutamine:fructose-6-phosphate amidotransferase (GFAT) is the rate-limiting enzyme for the HBP. *O*-GlcNAc transferase (OGT) adds UDP-GlcNAc to target protein serine and threonine residues, and *O*-GlcNAcase (OGA) removes *O*-GlcNAc from O-GlcNAcylated proteins. The balance between the enzyme activities of phosphofructokinase (PFK) and GFAT through regulating GFAT may be crucial to direct the pathways. G6P glucose 6-phosphate, F6P fructose 6-phosphate, FBP fructose 1,6-phosphate, GlcN6P glucosamine-6-phosphate, GlcNAc6P N-acetylglucosamine-6-phosphate, GlcNAc1P N-acetylglucosamine-1-phosphate, GAT acetyl-CoA:D-glucosamine-6-phosphate N-acetyltransferase, AGM phosphor-N-acetylglucosamine mutase, AGX1 UDP-GlcNAc pyrophosphorylase, GNK GlcNAc kinase. The graph was created with BioRender.com.

**Fig. 3:**
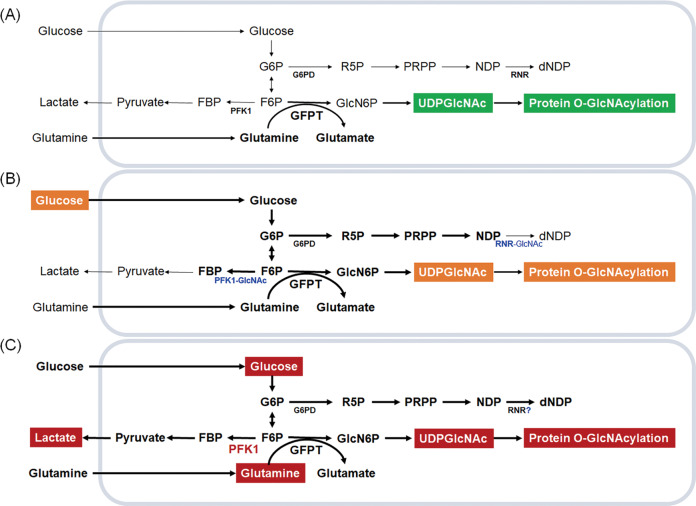
Availability and utilization of glucose and glutamine to generate uridine diphosphate-N-acetylglucosamine (UDP-GlcNAc) in different states in pancreatic cells. **A** During resting, approximately 2–5% of cellular glucose enters the hexosamine biosynthesis pathway (HBP) to generate the end product UDP-GlcNAc. **B** During adaptation, high glucose induces genome instability through O-linked-N-acetylglucosaminylation (O-GlcNAcylation) of phosphofructokinase 1 (PFK1) and ribonucleotide reductase catalytic subunit M1 (RRM1) to direct glycolysis and the pentose phosphate pathway (PPP) into the HBP. **C** During hyperproliferation, such as in pancreatic cancer cells, both high glucose and high glutamine are required through overactivation/overexpression of PFK1, shunting glycolysis into the PPP and HBP to generate energy and biosynthesis precursors to meet the needs of cancer cells. G6P glucose 6-phosphate, F6P fructose 6-phosphate, FBP fructose 1,6-phosphate, GFAT glutamine:fructose-6-phosphate amidotransferase, R5P ribose 5-phosphate, PRPP phosphoribosyl pyrophosphate, NDP nucleoside diphosphate, dNDP deoxynucleoside diphosphates, GlcN6P glucosamine-6-phosphate, UDP-GlcNAc uridine diphosphate-N-acetylglucosamine.

Another crucial target for rewiring glycolysis in the HBP is GFAT. GFAT, the first and rate-limiting enzyme of HBP, catalyzes glucosamine 6-phosphate by integrating glucose and glutamine metabolism. High levels of GFAT predict a poor prognosis in patients with PDAC [74]. GFAT1 depletion diminished high glucose-induced DNA damage and colony formation in pancreatic cells [10]. GFAT2 upregulation positively correlates with hyaluronan synthesis and epithelial-to-mesenchymal transition [75]. Overexpression of both GFAT1 and GFAT2 is associated with poor survival of PDAC (TGCA dataset, unpublished results). The bidirectional PFK2 isozymes (PFKFB1, PFKFB2, PFKFB3, and PFKFB4) and TP53-induced glycolysis and apoptosis regulator (TIGAR), a fructose 2,6 bisphosphatase that shapes the metabolic profile of cells by modulating HBP, have yet to be explored. Understanding how to balance PFK and GFAT enzymatic activities during adaptation and hyperproliferation may provide new insights into the modulation of HBP (Figs. 2, 3).

Since epigenetics plays a key role in regulating gene expression in different tissues and cell types [76], its contribution to the causal relationship of metabolite preference toward lower PFK activity in pancreatic cells is of great interest. Furthermore, ER stress may trigger HBP overactivation. The unfolded protein response (UPR)-HBP axis is partially triggered via GFAT1, a direct transcriptional target of a spliced form of X-box binding protein 1 (XBP1s), to protect cells under stress [77]. XBP1s also promotes pro-survival signaling during acinar cell differentiation [78]. In addition, ER stress sensors such as glutathione peroxidase 7 relieve ER oxidative stress by promoting 78 kDa glucose-regulated protein chaperone activity [79], linking the stress sensor to HBP activity.

Glucosamine-phosphate N-acetyltransferase 1 (GNPNAT1) is a key enzyme associated with glucose and fatty acid catabolism and UDP-GlcNAc biosynthesis. Although GNPNAT1 overexpression is associated with poor survival of patients with PDAC, the mechanisms underlying the link remain to be explored (TGCA dataset, unpublished results). Overexpression of phosphoacetylglucosamine mutase (PGM3, a phospho-N-acetylglucosamine mutase) has been linked to gemcitabine resistance. The PGM3 inhibitor FR054 synergizes with gemcitabine to suppress PDAC growth by promoting the UPR and inhibiting EGFR-AKT signaling [80]. The expression of UDP-N-acetylglucosamine phosphorylase (UAP1), GlcNAc kinase (GNK), OGT, or OGA was not associated with poor survival of PDAC (TGCA dataset, unpublished results). These results suggest that multiple intertwined factors may connect the regulation of HBP pathway.

## Nutrient sharing and competition: how *KRAS*-mutated cells prevail in competition

### Stress from the pancreatic cell microenvironment

The TME of PDAC is composed mainly of stroma with primary fibroblasts and immune cells [31, 81] and is a physical and oxidative stress source. Fibroblast-induced cell dysfunction may occur through extracellular matrix (ECM)-induced physical destruction, resulting in pancreatic fibrosis [82] and PDAC progression [83]. Those tissue injuries lead fibroblasts to produce extensive ECM to increase interstitial stresses [84]. The stresses in PDAC may exceed ten times of those observed in a normal pancreas [85, 86]. Although stromal components create a metabolic niche for cancer cells to maintain tumor survival, they might also restrain cancer progression [87–90].

Oxidative stress from TME can be resulted from nutrient imbalances. Consistent with this notion, metabolic imbalance-induced genomic instability and DNA damage may involve oxidative stress and DDR inefficiency. Using nucleotide supplements to reverse high glucose-induced DNA damage supports this potential [10, 62]. Interestingly, similar example is that BRCA2 DNA repair-associated (*BRCA2*)-deficient cells also experience endogenous oxidative stress-blocked mtDNA replication and stability, which could be ameliorated by the ROS scavenger N-acetylcysteine [48]. The redox shuttle enables NADPH transfer from the cytosol to the mitochondria to support cellular homeostasis. However, a severely inefficient DDR from the challenge of metabolite imbalance may lead to cell death. BCL-2 family proteins control cell death or differentiation primarily through the irreversible release of intermembrane space proteins, caspase activation, and apoptosis through direct interaction-regulated mitochondrial outer membrane permeabilization (MOMP). Aberrant oxidative stress alters the affinities and relative abundance of BCL-2 family proteins, affecting BCL-2 family protein interactions [46]. However, no consistent trends in ROS levels have been detected between two nontumorigenic pancreatic cell lines upon high glucose-induced DNA damage [10]. Other cellular antioxidants, including superoxide dismutases, glutathione S-transferases, glutathione peroxidases, and periaxin, may also protect against ROS-induced cell death, partly through nuclear factor erythroid 2–related factor 2 [91]. Personalized nutrient guidelines for restraining specific tumor growth will be an interesting subject to explore.

### PDAC associated microbiome: an enigma

The microbiota may be another risk factor for PDAC. Differences in oral, gut, and pancreatic microbiota have been reported among patients with PDAC. Oral microbiota dysbiosis may contribute to PDAC pathogenesis [92]. PDAC pathogenesis has been reported to link to an increased abundance of periodontal disease-associated *Porphyromonas gingivalis* and *Fusobacterium* sp. but reduced abundances of *Neisseria elongata* and *Streptococcus mitis* [93]. High levels of plasma antibodies against *P. gingivalis* have been correlated with a reduced risk of PDAC [94].

The interaction between microbiota and host, nutrient/drug efficacy, and toxicity directly alter or indirectly modify host physiology and pharmacodynamics. Current gut microbiome-cancer associations are witnessed in bacteria causing tumor progression and bacteria modulating antitumor immune responses [95, 96], which are partially dependent on cometabolites derived from the host and microbiota, such as short-chain fatty acids, bile acids, and indole derivatives, which are greatly influenced by nutrients [97]. For gut dysbiosis, an increased abundance of Bacteroidetes but a reduced abundance of Firmicutes in patients with PDAC have been found [98]. A lower α-diversity in the gut microbiome in patients with PDAC was detected. Increased abundance of *Veillonella*, *Klebsiella*, and *Selenomonas* species and lipopolysaccharide-producing bacteria but decreased abundance of *Bifidobacterium* species and butyrate-producing bacteria have been reported [99]. The association between *Helicobacter pylori*, a gastric pathogen that colonizes ~50% of individuals worldwide, and PDAC pathogenesis has been noted [100] but remains controversial.

The human tumor microbiome includes tumor type-specific intratumoral bacteria [101–103]. The α-diversity of the tumor microbiome was higher among individuals with increased long-term survival. An intratumoral microbiome signature (*Pseudoxanthomonas*-*Streptomyces*-*Saccharopolyspora*-*Bacillus clausii*) was associated with long-term patient survival [102]. Altered gut microbiome composition and changes in host processing of bacteria-derived metabolites may imply the link between diabetes and PDAC initiation and progression with geographical, racial, dietary, and lifestyle-related differences [104].

### *KRAS*-mutated cells prevail in competition under high-fat-induced inflammation

**Fig. 4:**
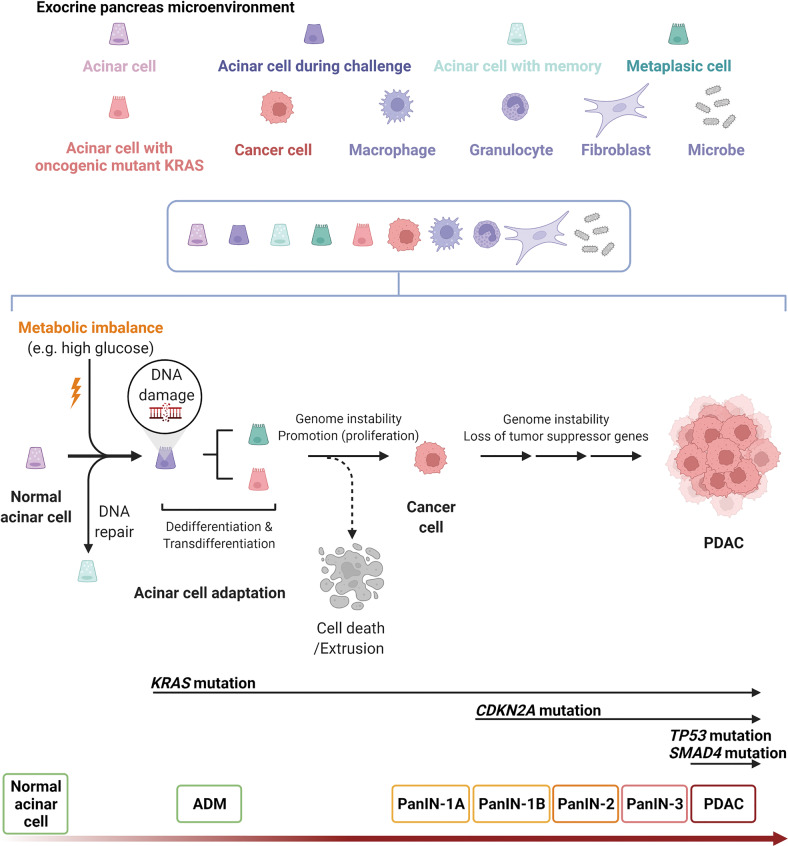
Normal acinar cells undergo sustained transcriptional and epigenetic reprogramming after challenging metabolic imbalance (e.g., high glucose). In response to DNA damage, cells may activate cell cycle checkpoints, process epigenetic and transcriptional programs or DNA repair (with epithelial memory), or undergo apoptosis when the damage is severe. Cells with adapted DNA mutation (e.g., oncogenic *KRAS* mutation) survived apoptosis and extrusion, with histologic and genetic progression, may transform into cancer cells and further grow as pancreatic ductal adenocarcinoma (PDAC) in adaptive to exocrine microenvironment-induced genome instability. Histologically, PDAC stems from microscopic abnormal acinar-to-ductal metaplasia (ADM) and pancreatic intraepithelial neoplasias (PanINs) of grades I–III to PDAC with increasing disorganization and nuclear abnormalities. High percentages of patients with PDAC carry somatic mutations, including KRAS proto-oncogene, GTPase (*KRAS*; ~95%) cyclin-dependent kinase inhibitor 2A (*CDKN2A*; ~90%), tumor protein 53 (*TP53*; ~70%), and SMAD family member 4 (*SMAD4*; ~55%). The pancreatic exocrine microenvironment is composed of exocrine cells at different stages, fibroblasts, immune cells, and microbes. The role of these microenvironmental components in adaptation, cancer initiation and progression remains to be elucidated. The graph was created with BioRender.com.

Not all pancreatic cells develop PanIN in genetically engineered mice with the *Kras*^*G12D*^ mutation, suggesting that additional factors are needed for the initial transformation. Figure 4 illustrates the current understanding of high glucose-triggered cancer initiation, which may be further promoted to become PDAC. Mutant cells are often recognized and passively eliminated from epithelial tissues through epithelial defense against cancer [105]. *Kras*-mutant cells drive metabolic reprogramming that enables them to rapidly grow and outcompete their adjacent regular counterparts to coexist with a large proportion of healthy cells in the preexisting general population [106]. High-fat diet feeding-induced inflammation promotes the coexistence of *Kras*-mutant cells with epithelia [107–109]. *Kras*^*G12D*^ maintains an irreversible ADM through MAPK constitutive signaling to protect against inflammation-induced tissue damage [110]. The evidence provides certain clues for how *KRAS*-mutated cancer cells may succeed in a nutrient competition.

### Functional genomics to uncover the metabolic dependence of PDAC

Studies on the in vivo metabolic dependence of PDAC have been challenging due to the complexity and heterogeneity of the TME. Although there are limitations regarding intercellular nutrient sharing, in vivo metabolism-focused CRISPR screening in PDAC is expected to determine the essential global metabolic dependence for PDAC tumor growth [111, 112]. Current findings revealed that tumor growth depends on the crosstalk between the immune system and heme biosynthesis [111]. Genetic or pharmacological inhibition of farnesyl diphosphate farnesyl transferase delayed tumor growth and promoted CD8^+^ T-cell infiltration through PI3K/AKT signaling, indicating the potential targeting of cholesterol biosynthesis and autophagy to combat PDAC [112]. A new platform for characterizing metabolic dependencies under distinct genetic drivers or different PDAC statuses, such as initiation and metastasis, will provide a breakthrough for the field.

## BOX1: Complexity of genetic alterations and metabolism in PDAC

In addition to the most frequent oncogenic somatic mutation *KRAS*, a high percentage of patients carry inactivating somatic mutations in the tumor suppressors cyclin-dependent kinase inhibitor 2 A (*CDKN2A*), tumor protein 53 (*TP53*), and SMAD family member 4 (*SMAD4*) [113–115], reinforcing their roles during PanIN-to-PDAC [116]. Other mutations, such as those in Gα protein subunits (such as *GNAS*), *MYC*, *TP53*, and *PTEN*, drive metabolic shifts in PDAC. *GNAS*-activating mutations increase lipid utilization and fatty acid oxidation to promote PDAC tumor progression [117]. *MYC* orchestrates metabolic reprogramming of PDAC progression through extrinsic and intrinsic factors via its natural role as a transcription factor [118–121]. *TP53* controls cellular metabolism by directly modulating different transcriptional programs, such as the autophagy network [122], or redox control through the p53 target TIGAR [123, 124]. Restoration of wild-type p53 induces α-ketoglutarate accumulation to increase chromatin accessibility and tumor suppression [125]. Loss of the tumor suppressor *PTEN* hyperactivates phosphoinositide-3-kinase (PI3K)–AKT signaling and metabolic processes such as glucose metabolism, *de novo* lipid synthesis, and redox balance [126] and cooperates with mutant *KRAS*-driven events in multiple PDAC models [127–130].

There is still much room for metabolic efforts for early diagnosis and prevention. Approximately 3–10% of patients with PDAC carry inherited germline mutations in genes such as BRCA1 DNA repair-associated (*BRCA1*), *BRCA2*, and ATM serine/threonine kinase [131]. The direct functional link of *BRCA1* and *BRCA2* to the DNA damage response was first demonstrated [132, 133]. The discovery of more sensitive poly(ADP-ribose) polymerase (PARP) inhibition in *BRCA*-mutant cancer cells has led to the development of new biomarker-driven synthetic lethal treatment strategies for different cancers [134, 135]. However, in a mouse model of PDAC initiation, loss of heterozygosity (LOH) of *BRCA2* while promoting chromosomal instability may not be essential for PDAC initiation [136]. Intriguingly, even in the presence of *KRAS* oncogenic mutations, *BRCA2* LOH inhibits tumor formation when wild-type *TP53* remains. *BRCA2* LOH can accelerate PDAC tumorigenesis only after *TP53* is mutated [137, 138]. PDAC metabolism shifts in response to the BRCAness phenotype remain to be elucidated. These findings postulated that the selected population sharing the BRCAness phenotype would be more sensitive to DNA damaging agents and DDR inhibitors. The appropriate subtype, therapeutic window, potential combination strategies, and functional differences of specific variants in the DDR pathway remain defined.

Approximately 5% of patients with PDAC carry *RB* mutations or deletions. *RB* was cloned and sequenced in the 1980s [139] and has been well characterized as a tumor suppressor for inhibiting the G0/G1 to S phase transition during cell cycle progression [140]. *RB* silences gene transcription by recruiting corepressor complexes, including histone deacetylases, to E2F transcription factors specifically targeting gene promotors [141]. Loss of *RB* inhibits glucose oxidation by directly inducing the expression of a glucose homeostasis sensor and modulator pyruvate dehydrogenase kinase 4 [141], promotes glutamine uptake via increased expression of the glutamine transporter and GLS1, perturbing redox homeostasis by reducing GSH levels [142]. However, the role and functional alteration of the loss of RB in PDAC development remain to be characterized.

## Concluding remarks

PDAC remains a difficult-to-treat cancer. Despite state-of-the-art comprehensive detection approaches, such as ultrasound, computed tomography scans, magnetic resonance imaging, and positron emission tomography scans, as well as treatment approaches such as surgery, radiation, chemotherapy, and immunotherapy, overall survival has not improved over the past several decades. Knowledge about metabolic dysregulation promoted by the RAS protein family, particularly mutant KRAS, has advanced substantially. Oncogenic KRAS mutations and their downstream reprogrammed metabolic pathways have been attractive therapeutic targets. Since metabolites such as glucose may trigger DNA damage and mutation through glucose metabolic reprogramming, a new niche for PDAC management has been emerged from these findings for early detection, prevention and treatment.

## Data Availability

There are no experimental datasets given that this is a review article prepared based on the literature review. All the data supporting the findings of this review are available from the corresponding author upon reasonable request.
